# Dasatinib Targets B-Lineage Cells but Does Not Provide an Effective Therapy for Myeloproliferative Disease in c-Cbl RING Finger Mutant Mice

**DOI:** 10.1371/journal.pone.0094717

**Published:** 2014-04-09

**Authors:** Johanna M. Duyvestyn, Samuel J. Taylor, Samantha A. Dagger, Marlene Orandle, Herbert C. Morse, Christine B. F. Thien, Wallace Y. Langdon

**Affiliations:** 1 School of Pathology and Laboratory Medicine, University of Western Australia, Crawley, Western Australia, Australia; 2 Infectious Disease Pathogenesis Section, Comparative Medicine Branch, National Institute of Allergy and Infectious Diseases, National Institutes of Health, Rockville, Maryland, United States of America; 3 Virology and Cellular Immunology Section, Laboratory of Immunogenetics, National Institute of Allergy and Infectious Diseases, National Institutes of Health, Rockville, Maryland, United States of America; B.C. Cancer Agency, Canada

## Abstract

This study aimed to determine whether the multi-kinase inhibitor dasatinib would provide an effective therapy for myeloproliferative diseases (MPDs) involving c-Cbl mutations. These mutations, which occur in the RING finger and linker domains, abolish the ability of c-Cbl to function as an E3 ubiquitin ligase and downregulate activated protein tyrosine kinases. Here we analyzed the effects of dasatinib in a c-Cbl RING finger mutant mouse that develops an MPD with a phenotype similar to the human MPDs. The mice are characterized by enhanced tyrosine kinase signaling resulting in an expansion of hematopoietic stem cells, multipotent progenitors and cells within the myeloid lineage. Since c-Cbl is a negative regulator of c-Kit and Src signaling we reasoned that dasatinib, which targets these kinases, would be an effective therapy. Furthermore, two recent studies showed dasatinib to be effective in inhibiting the *in vitro* growth of cells from leukemia patients with c-Cbl RING finger and linker domain mutations. Surprisingly we found that dasatinib did not provide an effective therapy for c-Cbl RING finger mutant mice since it did not suppress any of the hematopoietic lineages that promote MPD development. Thus we conclude that dasatinib may not be an appropriate therapy for leukemia patients with c-Cbl mutations. We did however find that dasatinib caused a marked reduction of pre-B cells and immature B cells which correlated with a loss of Src activity. This study is therefore the first to provide a detailed characterization of *in vivo* effects of dasatinib in a hematopoietic disorder that is driven by protein tyrosine kinases other than BCR-ABL.

## Introduction

c-Cbl is a RING finger-based E3 ubiquitin ligase that is highly expressed in hematopoietic cells where it functions as a negative regulator by directing the polyubiquitylation and degradation of Src family kinases (SFKs) and receptor tyrosine kinases such as c-Kit and FLT3 [1], [2]. The E3 ligase activity of c-Cbl is dependent on its linker and RING finger domains that associate with E2 ubiquitin conjugating enzymes to mediate the transfer of ubiquitin to c-Cbl-targeted tyrosine kinases. Mutations in the c-Cbl linker and RING finger domains have predominantly been identified in patients within the groupings of myelodysplastic syndromes and myelodysplastic/myeloproliferative neoplasms [3]–[10].

To investigate c-Cbl-associated malignancies we generated a c-Cbl RING finger mutant mouse that develops a severe myeloproliferative disease (MPD) progressing to lethal leukemia [11]. This mouse expresses an inactivating knock-in mutation in the RING finger domain (C379A) that disrupts the interaction with E2 conjugating enzymes therefore preventing E3 ligase activity [12]–[14]. The majority of the c-Cbl RING finger mutant mice succumb within a year with elevated white blood cell counts, splenomegaly, myeloid infiltration into peripheral organs and an expansion of multipotent progenitors (MPPs) and hematopoietic stem cells (HSCs) [11].

Analysis of hematopoietic progenitors and stem cells from c-Cbl(C379A) mice stimulated with stem cell factor (i.e. c-Kit ligand) or FLT3 ligand showed a marked enhancement of Akt and Erk activation suggesting that targeting c-Kit and/or FLT3 could provide effective therapies [11]. We recently found that the FLT3 inhibitor quizartinib (AC220) effectively suppresses MPD development in c-Cbl RING finger mutant mice [15]. Here we extend these studies to investigate dasatinib, a small molecule tyrosine kinase inhibitor that targets a variety of tyrosine kinases implicated in the pathophysiology of many types of tumors [16]. Among the most sensitive dasatinib targets are Abl, SFKs, and receptor tyrosine kinases c-Kit, platelet-derived growth factor receptor, c-Fms, and ephrin receptors [17]. The importance of investigating dasatinib in an animal model is highlighted by encouraging results where dasatinib inhibited the *in vitro* growth of human acute myeloid leukemia (AML) and juvenile myelomonocytic leukemia (JMML) cells with c-Cbl RING finger and linker domain mutations [18], [19]. In addition, c-Kit and FLT3 activate SFKs [20]–[24], and hyperactivation of SFKs occurs in c-Cbl(C379A) mice [25], further supporting the hypothesis that dasatinib may provide an effective treatment for leukemias associated with c-Cbl mutations.

In this study we find dasatinib to be ineffective for treating the MPD, or suppressing the expanded populations of HSCs and MPPs that are associated with c-Cbl RING finger mutant mice. However, dasatinib does have a rapid and profound effect in markedly reducing B-lineage cells, specifically targeting immature and pre-B cell populations.

## Materials and Methods

### Mice

The generation of c-Cbl(C379A) RING finger mutant mice (c-Cbl^A/−^) has previously been described [25]. Male C57BL/6.CD45.1 mice were purchased from the Animal Resource Centre (Canning Vale, Western Australia). C57BL/6.CD45.1 mice were lethally irradiated (2×5.5 Gy) at 9 weeks of age and repopulated by tail vein injection with 2×10^6^ bone marrow cells from 8–10 week old c-Cbl^A/−^ mutant mice. These mice were dosed at 18 weeks after transplantation.

### Ethics Statement

All mouse experiments were performed in strict accordance with the regulations and recommendations of the Animal Ethics Committee at the University of Western Australia. The Animal Ethics Approvals for this study are 100/786 and 100/1169.

### Dasatinib

Dasatinib was obtained from Bristol Myers-Squibb and LC laboratories (Woburn, MA). Stock solutions were prepared freshly each week in 80 mM Citric Acid, pH 2.1 and diluted each day in citrate buffer pH 3.1 for dosing. Mice were dosed with 0.2 ml of either dasatinib (as indicated in the results sections), or with citrate buffer vehicle, by oral gavage using 20G 1.5 inch feeding needles (Braintree, MA).

### Analysis of Peripheral Blood, Spleen and Bone Marrow Cells

Blood was collected from the tail vein and differential cell counts determined using a Hemavet HV950FS blood analyzer (Drew Scientific, Waterbury, CT). Bone marrow cells were taken from tibias and femurs of each mouse. Blood, bone marrow and spleen cells were analyzed by flow cytometry (FACS Canto, BD Biosciences) using monoclonal antibodies and procedures described below. Data was collected using FACSDiva version 6 software (BD Biosciences) and analyzed using FlowJo 9.3.1 software (Tree Star, Inc., Ashland, OR).

### Flow Cytometry Antibodies and Procedures

All antibodies are from BD Biosciences, except where noted otherwise. CD3-biotin, CD11b-FITC -PE -PE-Cy7 -biotin, CD16/32-PE, CD19-PE, CD24-PE-Cy7, CD34-FITC, CD43-PE, CD48-FITC, CD95-PE, CD150-PE-Cy7, c-Kit-APC -FITC (eBioscience), Sca-1-PE – PE-Cy7 (eBioscience), B220-biotin, FLT3-PE (eBiosciences), Gr-1-FITC -biotin, IgM-APC, IgD-FITC (eBioscience), IL-7R-APC -biotin, TER119-biotin, GL7-FITC and TCR-APC. Cells incubated with biotinylated antibodies were treated with streptavidin conjugated with APC-Cy7 (BD Biosciences). Multipotent progenitors (MPPs) as FLT3^+^ LSK cells, common lymphoid progenitors (CLPs) as Lin^−^, IL-7R^+^ Sca-1^lo^ c-Kit^lo^; granulocyte macrophage progenitors (GMPs) as Lin^−^, CD16/32^hi^, CD34^+^, Sca1^−^, c-Kit^+^; common myeloid progenitors (CMPs) as Lin^−^, CD16/32^lo^, CD34^+^, Sca1^−^, c-Kit^+^; and megakaryocyte erythroid progenitors (MEPs) as Lin^−^_,_ CD34^−^, CD16/32^−^, Sca-1^−^,c-Kit^+^. Pre-B, pro-B and prepro-B cells were determined by CD24 and CD43 staining of B220^+^, IgM^−^ bone marrow cells.

### Histopathology

Spleens were fixed in Bouin fixative for 48 hours followed by two washes in 70% ethanol. Processing was carried out using a Leica TP 1020 processor, and paraffin-embedded sections (4–5 μm) were stained with hematoxylin and eosin (H&E). Photomicrographs were taken using an Olympus BX51 microscope (4×/0.16 and 20×/0.70 objectives) and an Olympus DP70 camera. Blood films were examined using an Olympus BX51 microscope (60×/0.09 objective) and photographed with an Olympus SIS 3VCU camera.

### Immunoblotting

Lysates from peripheral white blood cells and bone marrow cells were prepared in SDS sample buffer and examined by immunoblotting with the following antibodies: anti-phosphotyrosine (4G10, Millipore), anti-phospho-Src(Y416) (D49G4, Cell Signaling Technology), anti-Lyn (Santa Cruz), and anti-GAPDH (Abcam). Horseradish peroxidase-conjugated anti-mouse and anti-rabbit IgG antibodies were obtained from Cell Signaling Technology.

### Statistical Analyses

To validate significance we used unpaired two-sided Student’s *t*-tests (Prism 5, GraphPad Software). P values less than 0.05 were considered statistically significant. All statistical data are presented as means ± standard errors.

## Results

### Dasatinib Reduces Lymphocytes Numbers but does not Suppress the MPD in c-Cbl RING Finger Mutant Mice

Since homozygous c-Cbl RING finger mutant mice die *in utero* we analyze c-Cbl(C379A) knock-in mice that express a single mutant c-Cbl RING finger allele and a c-Cbl null allele, i.e. c-Cbl^A/−^ mice, that are generated from matings between c-Cbl^−/−^×c-Cbl^A/+^ mice [25].

To determine the effectiveness of dasatinib for treating c-Cbl-associated MPD we established a cohort of c-Cbl^A/−^ mice aged 8–9 months that was dosed once daily for 4 weeks with 15 mg/kg of dasatinib or vehicle. This dose was chosen based on previous mouse studies and recommendations from Bristol Myers-Squibb to approximate daily doses given to chronic myeloid leukemia patients [16].

**Figure 1:**
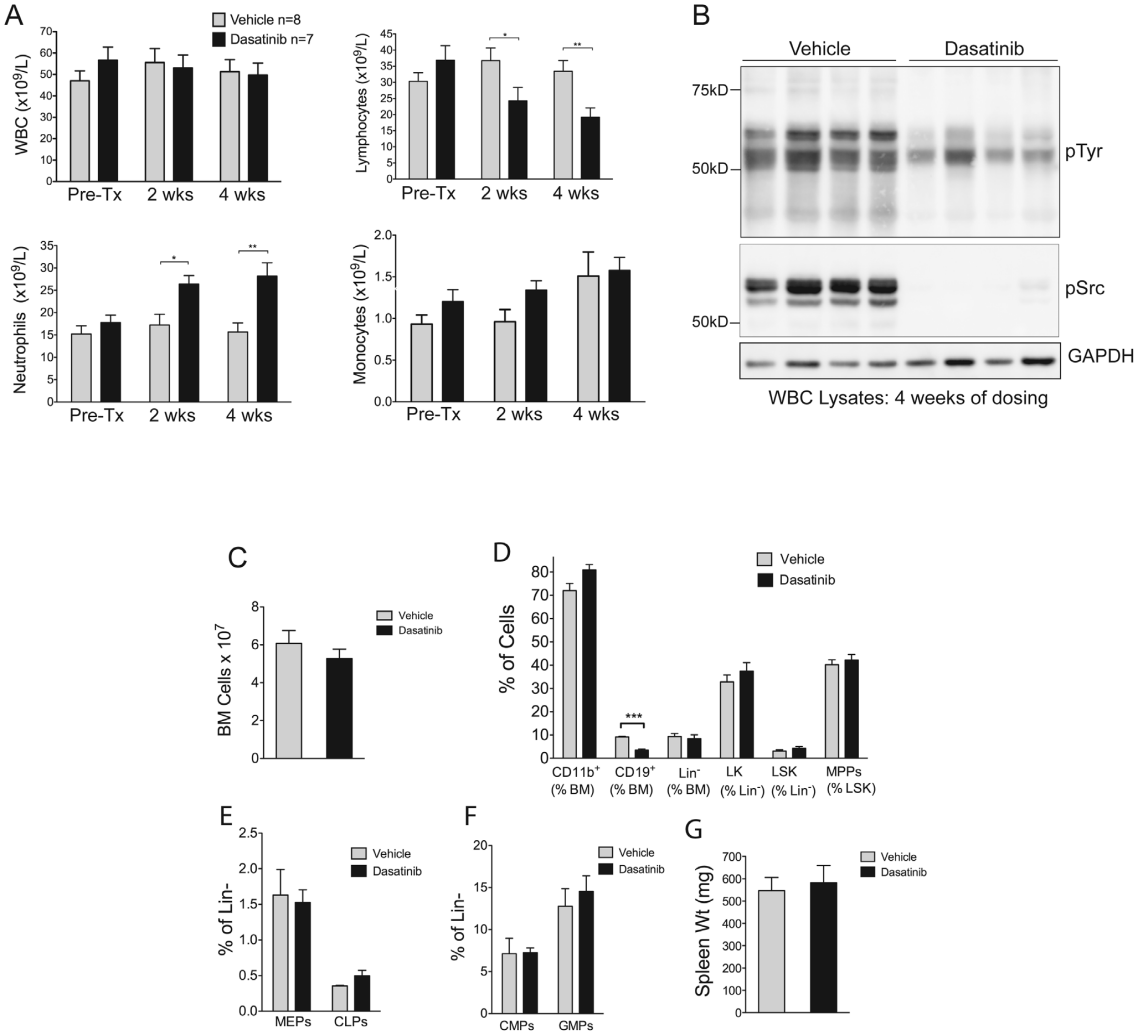
Dosing c-Cbl RING finger mutant mice with dasatinib results in a reduction of lymphocytes and a corresponding increase in neutrophils. c-Cbl^A/−^ mice aged 8–9 months were bled 6 days before dosing (Pre-Tx), and after 2 and 4 weeks of daily dosing with 15 mg/kg of dasatinib or vehicle. (A) Numbers of white blood cells (WBC), lymphocytes, neutrophils and monocytes. The counts are expressed as means ± standard errors. *P<0.05, **P<0.01, using unpaired Student’s *t* test. (B) WBC lysates prepared from mice dosed for 4 weeks with vehicle or dasatinib were immunoblotted with the indicated antibodies. (C) Analysis of mice after 4 weeks of dosing showing the numbers of nucleated bone marrow cells, (D) the proportion of CD11b^+^; CD19^+^; lineage negative (Lin^−^); Lin^−^, c-Kit^+^ (LK); Lin^−^, Sca-1^+^, c-Kit^+^ (LSK) cells; and multi-potent progenitors (MPPs: defined as FLT3^+^ LSK cells), expressed as percentages of the indicated populations. (E) Megakaryocyte erythroid progenitors (MEPs) and common lymphoid progenitors (CLPs), and (F) common myeloid progenitors (CMPs) and granulocyte-macrophage progenitors (GMPs) expressed as percentages of Lin^−^ bone marrow cells. The bone marrow data is from 4 dasatinib and 4 vehicle treated mice, and the results are expressed as means ± standard errors. ***P<0.001 using the unpaired Student’s *t* test. (G) Spleen weights from 4 vehicle and 4 dasatinib treated mice after 4 weeks of dosing.

Mice were bled 6 days before dosing commenced, and after 2 and 4 weeks of dosing, to determine peripheral white blood cell (WBC), neutrophil, lymphocyte and monocyte numbers (Fig. 1A). Before treatment the assigned vehicle and dasatinib groups had WBC counts averaging 47 and 57×10^9^/L respectively. For comparison, c-Cbl^+/−^ littermates at 8–9 months of age have WBC counts of 10–15×10^9^/L [11]. We found that dosing with dasatinib did not result in a significant reduction in total WBC numbers. Surprisingly however dasatinib significantly reduced the numbers of lymphocytes but caused an equivalent increase in neutrophils (Fig. 1A). Monocyte numbers remained unaffected (Fig. 1A).

To determine whether dasatinib treatment inhibited protein tyrosine phosphorylation we prepared WBC lysates from mice dosed for 4 weeks (Fig. 1B). Immunoblotting with anti-phosphotyrosine and anti-phospho-Src showed that dasatinib was very effective in causing a marked reduction in total phosphotyrosine and phospho-Src levels compared to vehicle treated mice. (Fig. 1B).

### Hematopoietic Progenitor and Stem Cell Numbers are Unaffected by Dasatinib

To determine whether dasatinib affects cell populations in the bone marrow we analyzed vehicle and dasatinib treated mice after 4 weeks of dosing. No significant differences were observed between the two groups in the total number of nucleated bone marrow cells (Fig. 1C). However flow cytometry analysis with anti-CD19 antibodies revealed a significant reduction in B-lineage cells following dosing with dasatinib (Fig. 1D). In contrast, there were no changes in the percentages of CD11b^+^ myeloid-lineage cells, with similar proportions of Gr-1^+^ and Gr-1^−^ cells within this population (data not shown). Similarly the percentages of lineage negative (Lin^−^), Lin^−^ c-Kit^+^ (LK), Lin^−^ Sca-1^+^ c-Kit^+^ (LSK), or multipotent progenitor (MPP) cells (i.e. FLT3^+^ LSK cells) were unaffected (Fig. 1D). Dasatinib was also found to have no effect on megakaryocyte erythroid progenitors (MEPs), common lymphoid progenitors (CLPs) (Fig. 1E), common myeloid progenitors (CMPs) or granulocyte macrophage progenitors (GMPs) (Fig. 1F). In addition, 4 weeks of dosing did not significantly reduce the splenomegaly that characterizes c-Cbl^A/−^ mice (Fig. 1G).

### High Doses of Dasatinib Reduce WBC Counts through a Loss of B lymphocytes

**Figure 2:**
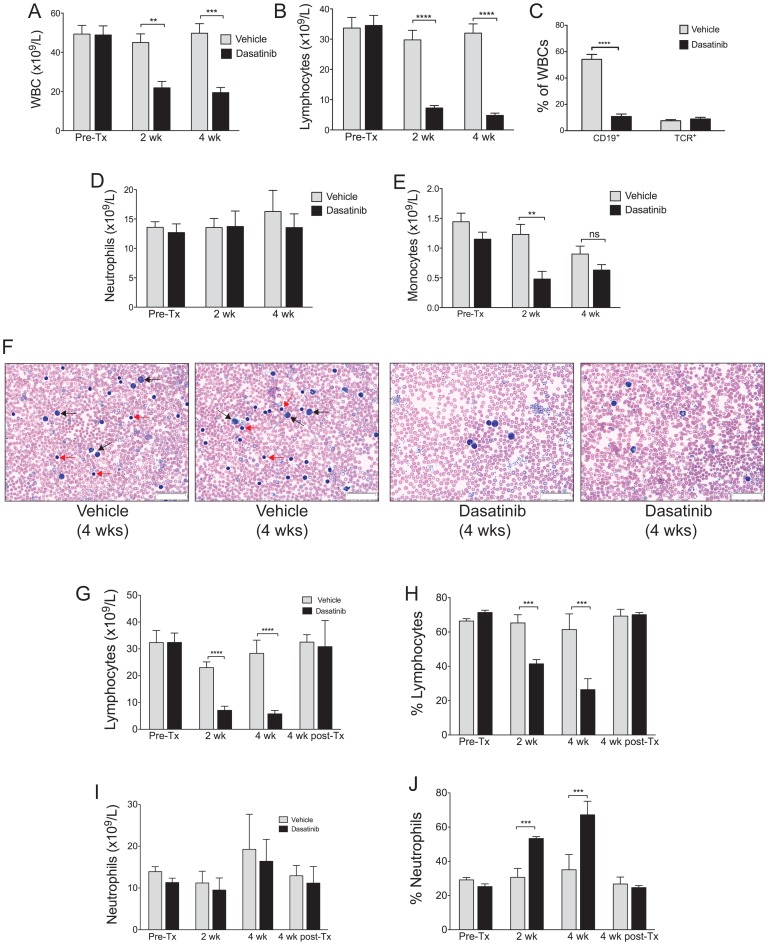
High dose dasatinib treatment results in a significant reduction of B lymphocytes in the blood. c-Cbl RING finger mutant mice aged 8–9 months were dosed daily with 30 mg/kg (am) +50 mg/kg (pm) of dasatinib or vehicle, and bled before treatment (Pre-Tx), and after 2 and 4 weeks of treatment. Differential blood counts from 9 vehicle and 7 dasatinib treated mice were determined by Hemavet analysis. Shown are (A) total WBC numbers and (B) lymphocyte numbers. (C) WBCs were analyzed by flow cytometry to determine the percentage of B-lineage cells, by anti-CD19 staining, and the percentage of T cells, by anti-T cell receptor staining. (D) Numbers of neutrophils and (E) monocytes. (F) Blood films from 2 vehicle and 2 dasatinib treated mice following 4 weeks of dosing illustrate the loss of lymphocytes in the dasatinib-treated mice. Lymphocytes are indicated by red arrows, and myeloid cells by black arrows, in the blood films from the two vehicle treated mice. The images were acquired at room temperature using an Olympus BX51 microscope with a 60×/0.09 objective and photographed with a SIS 3VCU Olympus digital camera. Scale bar = 50 μm. Lymphocyte numbers (G) and percentages (H) in the blood return to pre-treatment levels 4 weeks after ceasing dasatinib treatment. Neutrophil numbers remain unaltered (I) but the high percentages (J) returned to normal 4 weeks after dasatinib dosing ceases. Mice were bled from the tail vein before treatment (Pre-Tx), after 2 and 4 weeks of treatment, and following 4 weeks without treatment (i.e. post-Tx). Numbers and percentages were determined by Hemavet differential counting, and are from 4 vehicle and 3 dasatinib treated mice. The results are expressed as means ± standard errors. **P<0.01, ***P<0.001, ****P<0.0001 using unpaired Student’s *t* test.

The minimal effects of dosing c-Cbl^A/−^ mice with 15 mg/kg was surprising given the promising findings from *in vitro* studies examining the inhibitory effects of dasatinib on cells from AML and JMML patients with c-Cbl mutations [18], [19]. We therefore examined whether increasing the dose of dasatinib could provide an effective therapy. A daily regimen of 30 mg/kg +50 mg/kg 8 hours apart was chosen following advice from BMS and studies demonstrating mice could tolerate doses up to 150 mg/kg [26]–[30]. Although these doses represent drug exposures approximately seven fold higher than those typically given to CML patients they were chosen to maximize the chances of affecting the MPPs and HSCs that drive the MPD. c-Cbl^A/−^ mice aged 7–8 months were dosed with either vehicle or dasatinib, with the average WBC counts before treatment being 49.3 and 48.8×10^9^/L for vehicle and dasatinib groups respectively. Blood taken after 2 and 4 weeks of dosing showed a significant decrease in total WBC counts in the dasatinib group (Fig. 2A), which was predominantly due to a loss of lymphocytes (Fig. 2B). Analysis by flow cytometry showed this was caused by a loss of B lymphocytes, but not T lymphocytes which were unaffected (Fig. 2C). Neutrophil numbers were unaffected (Fig. 2D), and while monocyte numbers were slightly reduced after 2 and 4 weeks of dosing, this effect was only statistically significant at 2 weeks (Fig. 2E). Representative blood films from 2 vehicle and 2 dasatinib-treated mice illustrate the effect of dasatinib in promoting the loss of lymphocytes (red arrows), resulting in a corresponding increase in the proportion of large myeloid lineage cells (black arrows) (Fig. 2F).

These experiments also involved dosing normal littermates (i.e. c-Cbl^+/−^ mice); 3 with vehicle and 3 with dasatinib (Fig. S1). We found very similar effects to that observed with c-Cbl^A/−^ mice where dasatinib caused a marked loss of B lymphocytes, but not T lymphocytes (Fig. S1C). Similarly, dasatinib did not significantly affect neutrophil numbers in the blood (Fig. S1D). Monocyte numbers, although reduced after 2 weeks of dasatinib dosing, did not show a significant difference by 4 weeks (Fig. S1E).

c-Cbl^A/−^ mice that were not culled after 4 weeks of dosing were left untreated for a further 4 weeks. By this time lymphocyte numbers and percentages returned to levels equivalent to the vehicle-treated group (Fig. 2G and H). Consistent with this the high percentage of neutrophils in the blood returned to vehicle-treated levels 4 weeks after dosing ceased (Fig. 2J).

### High doses of Dasatinib do not Reduce Hematopoietic Progenitors or Stem Cells

**Figure 3:**
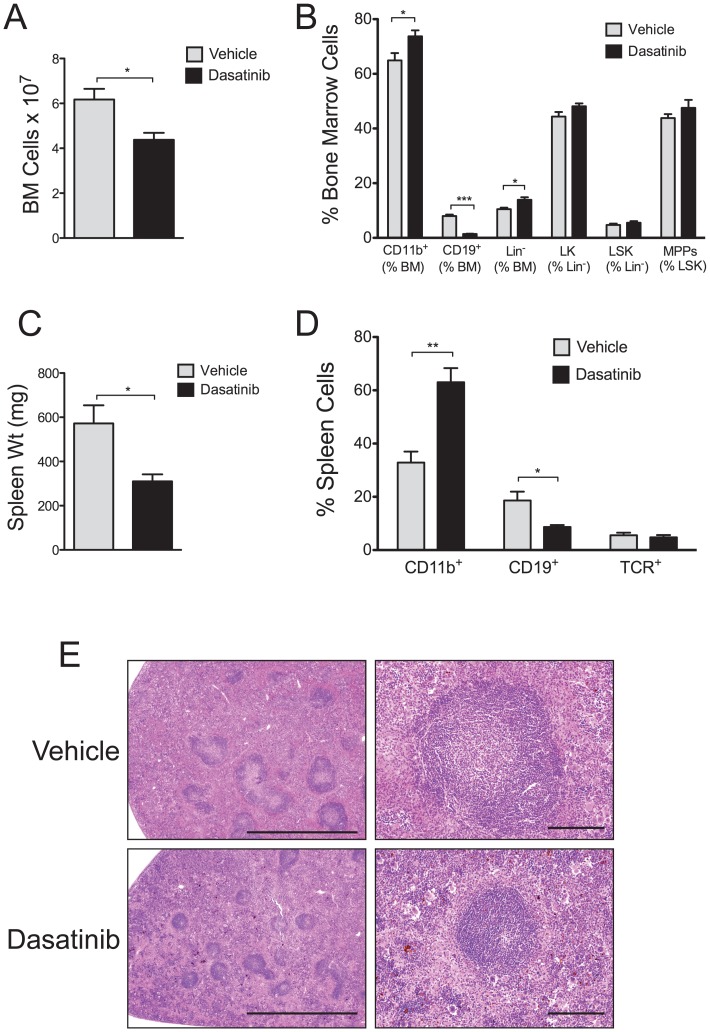
High dose dasatinib treatment markedly reduces the numbers of B-lineage cells. c-Cbl RING finger mutant mice aged 8–9 months were dosed daily with 30 mg/kg (am) +50 mg/kg (pm) of dasatinib or vehicle, and analyzed after 4 weeks. (A) Numbers of nucleated bone marrow cells from each group. (B) Bone marrow cells analyzed by flow cytometry to determine the percentage of CD11b^+^ myeloid-lineage cells; CD19^+^ B-lineage cells; lineage negative cells (Lin^−^); c-Kit^+^ lineage negative (LK) cells; Lin^−^ Sca-1^+^ c-Kit^+^ (LSK) cells; and multi-potent progenitors (MPPs). (C) Spleen weights from each treatment group. (D) Spleen cells analyzed by flow cytometry to determine percentages of CD11b^+^, CD19^+^ and T cell receptor^+^ (TCR) cells. All of the above data are from 4 dasatinib and 4 vehicle treated mice. The results are expressed as means ± standard errors. *P<0.05, **P<0.01, ***P<0.001 using the unpaired Student’s *t* test. (E) H&E stained sections of spleens showing the effects of dasatinib in markedly reducing the size of the follicles in the white pulp. The images were acquired at room temperature using an Olympus BX51 microscope. Photomicrographs were taken at 40× and 200× magnification with 4×/0.16 and 20×/0.70 objective lenses using an Olympus DP70 digital camera. Scale bars are 2 mm and 200 μm respectively.

Four weeks of dosing c-Cbl^A/−^ mice with 30+50 mg/kg of dasatinib resulted in a small but significant reduction in bone marrow cellularity (Fig. 3A). Flow cytometry analysis showed a marked reduction in CD19^+^ cells, and an increase in the proportion of CD11b^+^ cells consistent with findings in the peripheral blood (Fig. 3B). Very similar effects of dasatinib were also evident in the bone marrow of c-Cbl^+/−^ littermates (Fig. S2A and B). Further analysis of c-Cbl^A/−^ mice showed that the percentage of Lin^−^ cells was slightly increased, but no significant effect was observed in the proportion of LK, LSK or MPP cells (Fig. 3B), a surprising result given dasatinib’s ability to inhibit c-Kit and the *in vivo* requirement of these cells for c-Kit signalling [31].

The higher doses of dasatinib reduced splenomegaly (Fig. 3C) and, as seen with blood and bone marrow, the most notable effect was the loss of B cells and the corresponding increase in the proportion of myeloid lineage cells (Fig. 3D). Similar effects were also seen in c-Cbl^+/−^ littermates (Fig. S2C and D). The mechanism for the increase in the proportion of myeloid lineage cells remains to be resolved, however we did not detect an increase in either GM-CSF or G-CSF in the serum of dasatinib-treated mice that could account for a compensatory response (data not shown). Furthermore the loss of B cells may not fully account for the reduction in spleen size, however analysis of H&E stained sections shows the effect of dasatinib in reducing the size of follicles in the white pulp of the spleen (Fig. 3E). Although the reduction in the size of the splenic follicles is a likely contributing factor in decreasing the size of the spleens, further investigation is required to identify whether other lineages may also be affected.

Daily dosing of mice with 30+50 mg/kg of dasatinib caused side effects including laboured breathing and listlessness, and 2 of the 9 c-Cbl^A/−^ mice and 1 of the 3 c-Cbl^+/−^ succumbed. In contrast no ill effects were evident in the vehicle group.

### Dasatinib Markedly Reduces Phospho-Src Levels and pre-B cell Numbers in the Bone Marrow

**Figure 4:**
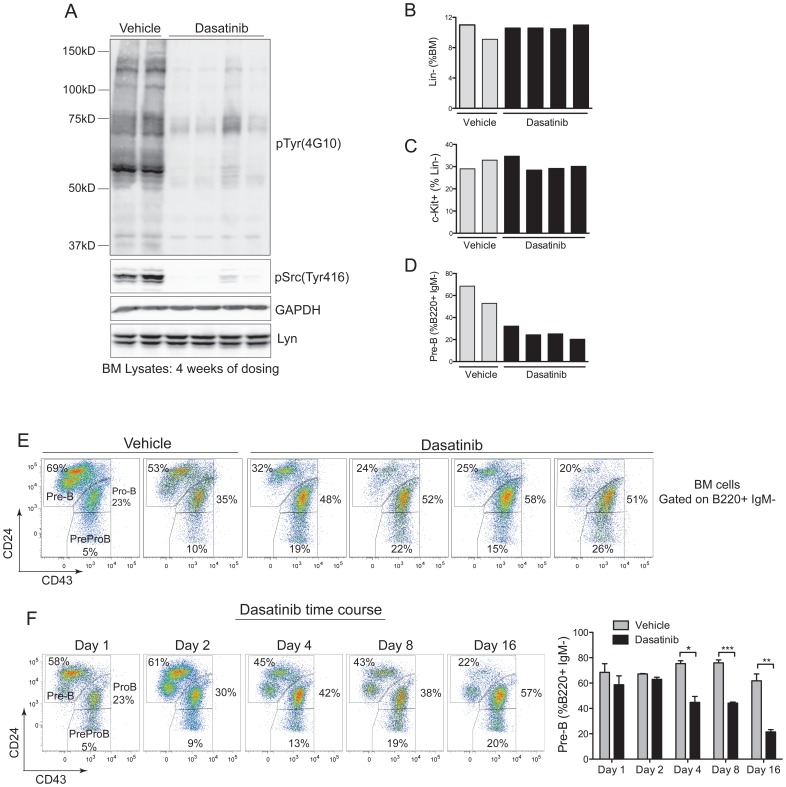
Dasatinib markedly reduces phosphotyrosine levels and pre-B cell numbers in the bone marrow. (A) Total bone marrow cell lysates from 2 vehicle and 4 dasatinib (15 mg/kg twice daily) treated c-Cbl^A/−^ mice following 4 weeks of dosing were immunoblotted with the indicated antibodies. Bone marrow cells from the 6 mice were phenotyped by flow cytometry to determine (B) the percentage of lineage negative cells, (C) percentage of c-Kit^+^ Lin^−^ cells and (D) percentage of pre-B cells, i.e. B220^+^ IgM^−^ CD24^+^ CD43^−^ cells. (E) Flow cytometry profiles showing the loss of pre-B cells in the dasatinib-treated mice. (F) B6.CD45.1 mice repopulated with c-Cbl^A/−^ bone marrow were dosed twice daily with 15 mg/kg of dasatinib for the indicated number of days. Bone marrow cells were analyzed to determine when the loss of pre-B cell became evident, with the data showing a significant loss of pre-B cells by day 4 of dosing. The results are from 3 mice per time point and treatment, and are expressed as means ± standard errors. *P<0.05, **P<0.01, ***P<0.001 using the unpaired Student’s *t* test.

To further investigate the effects of dasatinib in the bone marrow we dosed c-Cbl^A/−^ mice with 15 mg/kg of dasatinib or vehicle twice daily for 4 weeks, a regimen less likely to compromise the health of the mice and providing doses comparable to those used for treating CML patients. Analysis of bone marrow lysates by immunoblotting showed that dasatinib markedly reduced total tyrosine phosphorylation and phospho-Src (Fig. 4A). However, even with this marked loss of protein phosphorylation, no decrease was evident in lineage negative or c-Kit^+^ populations (Fig. 4B and C). In contrast, dasatinib markedly reduced the proportion of pre-B cells in the bone marrow (Fig. 4D and E), a subpopulation of B220^+^ IgM^−^ cells that are characterized as CD24^hi^ and CD43^lo^ [32].

To determine the kinetics of dasatinib’s effect on pre-B cells we dosed mice over a 16-day period (15 mg/kg twice daily), and analyzed mice on days 1, 2, 4, 8 and 16 (Fig. 4F). To obtain sufficient mice for this experiment we established B6.CD45.1 mice repopulated with c-Cbl^A/−^ bone marrow cells. We found that the loss of pre-B cells from the marrow was evident by day 4, and by day 16 pre-B cells were reduced to levels equivalent to those seen after 4 weeks of dosing (Fig. 4F).

**Figure 5:**
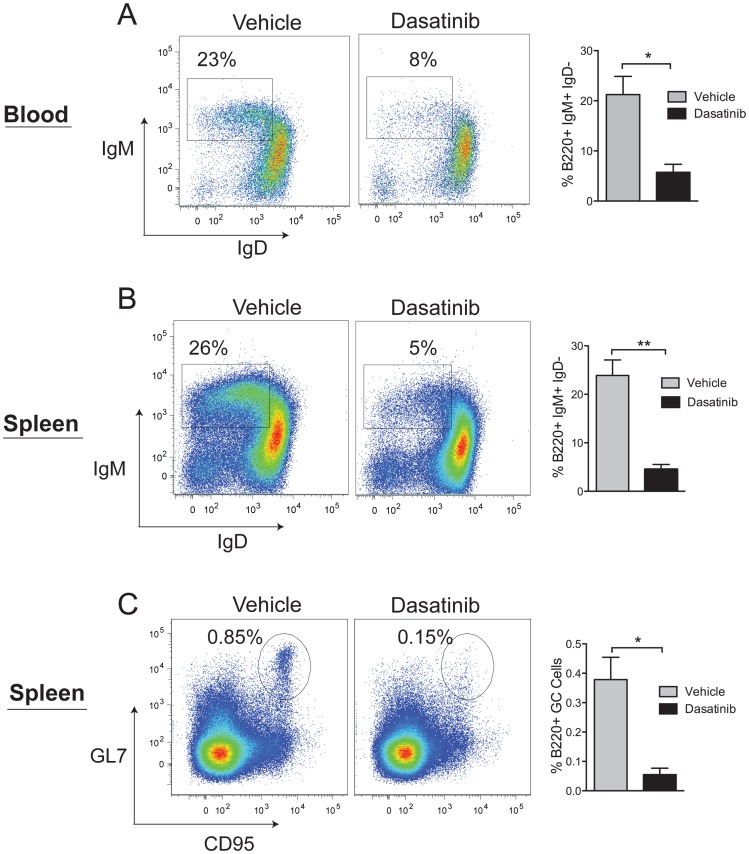
Dasatinib targets immature and germinal center B cells. B6.CD45.1 mice repopulated with bone marrow cells from a c-Cbl^A/−^ mouse were dosed twice daily with 15 mg/kg of dasatinib for 16 days. WBCs and spleen cells were analyzed by flow cytometry to identify B cell populations that are most sensitive to dasatinib. Shown are representative flow cytometry profiles and data from 3 vehicle and 3 dasatinib treated mice. (A) B220^+^ WBCs and (B) B220^+^ spleen cells were analyzed for the expression of IgM and IgD to identify immature and mature B cells. The gates show immature IgM^+^ IgD^−^ B cells. (C) B220^+^ spleen cells analyzed for the expression of GL7 and CD95 to identify germinal center (GC) B cells. The results are expressed as means ± standard errors. *P<0.05, **P<0.01 using the unpaired Student’s *t* test.

Further analysis of B cells in the blood and spleen was carried out at day 16. The blood showed dasatinib specifically targeted immature IgM^+^ IgD^−^ B cells, whereas the more mature IgD^+^ cells appeared unaffected (Fig. 5A). Similarly, in the spleen, immature IgM^+^ IgD^−^ transitional (T1) B cells were most susceptible to dasatinib (Fig. 5B), a result consistent with requirements for B cell receptor (BCR) signaling and SFK activation [33], [34]. The effect of dasatinib in reducing the splenic follicle size (Fig. 3E) prompted an investigation of germinal center (GC) cells, a rapidly proliferating population of B cells that is dependent on Lyn kinase signaling [35]–[37]. We found that dasatinib caused a significant reduction of B220^+^ cells expressing the GC markers CD95 and GL7 (Fig. 5C), a finding consistent with the notion that dasatinib would affect highly proliferative B-lineage cells that are reliant on BCR engagement and SFK activation.

## Discussion

Mutations that abolish the E3 ligase activity of c-Cbl cause hypersensitivity to growth factor stimulation leading to enhanced and sustained activation of protein tyrosine kinases. This prominent effect on cell signaling is very likely an important factor that contributes to the development of hematopoietic malignancies associated with c-Cbl RING finger and linker domain mutations. Therefore, targeting these aberrantly activated protein tyrosine kinases with small molecule inhibitors is an approach that could provide an effective therapy. Furthermore, hematopoietic malignancies associated with c-Cbl mutations, both in humans and in c-Cbl^A/−^ mice, are disorders of hematopoietic stem cells (HSCs), and therefore the successful targeting of these cells is an important goal. Indeed HSCs from c-Cbl^A/−^ mice are clearly perturbed as evidenced by their potent ability to out-compete wild-type HSCs in transplantation studies, and through the deregulated expression of many genes [11], [25], [38].

We recently found that the FLT3 kinase inhibitor quizartinib suppressed MPD development in c-Cbl RING finger mutant mice by markedly reducing the number of FLT3^+^ MPPs. However HSCs were not affected and a resurgence of disease occurred when treatment ceased [15]. We therefore hypothesized that dasatinib may provide an effective therapy by targeting c-Kit^+^ HSCs. In this study we demonstrated that dasatinib did not reduce the HSC populations, and as a consequence it did not provide an effective therapy. Thus we conclude that dasatinib as a monotherapy may not be appropriate for patients with c-Cbl mutations. This was disappointing in view of promising *in vitro* studies with dasatinib demonstrating growth inhibition of cells isolated from AML and JMML patients with c-Cbl RING finger and linker domain mutations [18], [19].

A key factor mediating the effect of dasatinib in the aforementioned *in vitro* studies was the inhibition of SFKs that are activated from highly responsive granulocyte macrophage-colony stimulating factor (GM-CSF) receptors. Since myeloid expansion and GM-CSF hyper-responsiveness are prominent features of the c-Cbl RING finger mutant mouse [11] (and unpublished data), it was surprising that dasatinib did not reduce this population. It is possible that compensatory responses through an abnormal induction of growth factors could account for this, although no increase in either GM-CSF or G-CSF was evident in the serum of dasatinib-treated mice (data not shown). However, compensatory responses are well characterized in humans and mice treated with FLT3 inhibitors [15], [39], so further analyses may reveal similar responses in animals treated with dasatinib. The contrasting outcomes between this present study and recent *in vitro* studies with dasatinib highlight the added complexities of animal models and underscore their relevance for studying anti-cancer treatments.

A prominent effect of dosing c-Cbl RING finger mutant mice with dasatinib was the marked reduction of B-lineage cells. Pre-B cells and immature IgM^+^ B cells were the most affected, and dasatinib’s marked inhibition of SFKs appears to be a major causative factor (Figs. 1 and 4). Furthermore, although our studies show that B-lineage cells from c-Cbl^+/−^ littermates are also effectively targeted by dasatinib (Fig. S1 and S2), a more detailed study is required to determine how extensively our findings with c-Cbl^A/−^ knock-in mice can be generalized to wild-type mice.

An inhibitory effect on B cells is consistent with three recent studies where dasatinib provided an effective therapy in a mouse model of Burkitt lymphoma [40], induced apoptosis of B cells cultured from chronic lymphocytic leukemia (CLL) patients [41] and, in a phase II study, had activity in relapsed and refractory CLL patients [42]. Thus, in addition to characterizing the specific SFKs in B cells that are targeted by dasatinib, additional consideration should be given for its use in treating B cell malignancies, and to monitor B cell numbers and function in Philadelphia positive CML patients who are treated with dasatinib.

## Conclusions

In summary, the findings from this study are in marked contrast to our previous work that showed the effectiveness of the FLT3 inhibitor quizartinib in treating the MPD of c-Cbl RING finger mutant mice [15]. Thus we conclude that leukemia patients with c-Cbl mutations should be considered for treatment with quizartinib rather than dasatinib. Additionally we have uncovered important new facets of dasatinib in an animal model that is not driven by BCR-ABL by revealing its inability suppress hematopoietic progenitors or myeloid lineage cells yet promoting a marked reduction of immature B-lineage cells.

## Supporting Information

Figure S1 High dose dasatinib treatment results in a significant reduction of B lymphocytes in the blood of normal c-Cbl^+/−^ littermates. c-Cbl^+/−^ mice aged 8–9 months were dosed daily with 30 mg/kg (am) +50 mg/kg (pm) of dasatinib or vehicle, and bled before treatment (Pre-Tx), and after 2 and 4 weeks of treatment. Differential blood counts from 3 vehicle and 3 dasatinib treated mice were determined by Hemavet analysis. Shown are (A) total WBC numbers and (B) lymphocyte numbers. (C) WBCs were analyzed by flow cytometry to determine the percentage of B-lineage cells, by anti-CD19 staining, and the percentage of T cells, by anti-T cell receptor staining. (D) Numbers of neutrophils and (E) monocytes. By 4 weeks of dosing one of the dasatinib-treated mice had died, therefore only two dasatinib-treated mice were available for analysis at this time point. The results are expressed as means ± standard errors. *P<0.05, **P<0.01, ***P<0.001 using the unpaired Student’s *t* test.
(TIF)

Figure S2 High dose dasatinib treatment markedly reduces B-lineage cells in the bone marrow and spleen of normal littermates. c-Cbl^+/−^ mice aged 8–9 months were dosed daily with 30 mg/kg (am) +50 mg/kg (pm) of dasatinib or vehicle, and 2 mice from each group were analyzed after 4 weeks. Bone marrow cells were analyzed by flow cytometry to determine the percentage of (A) CD19^+^ B-lineage cells and (B) CD11b^+^ myeloid cells. The results showed that dasatinib caused a marked reduction in B-lineage cells and a corresponding increase in the proportion of myeloid cells. (C) Analysis of spleen cells from these mice showed that dasatinib caused a large reduction in CD19^+^ B cells, whereas T cells were not markedly affected. (D) CD11b analysis showed that dasatinib caused an increase in the proportion of myeloid cells in the spleen.
(TIF)
