# Supplementary figures and images for: Dasatinib Targets B-Lineage Cells but Does Not Provide an Effective Therapy for Myeloproliferative Disease in c-Cbl RING Finger Mutant Mice
Source: PLoS One. 2014 Apr 9;9(4):e94717. doi: 10.1371/journal.pone.0094717 (PMC3981816; doi:10.1371/journal.pone.0094717)

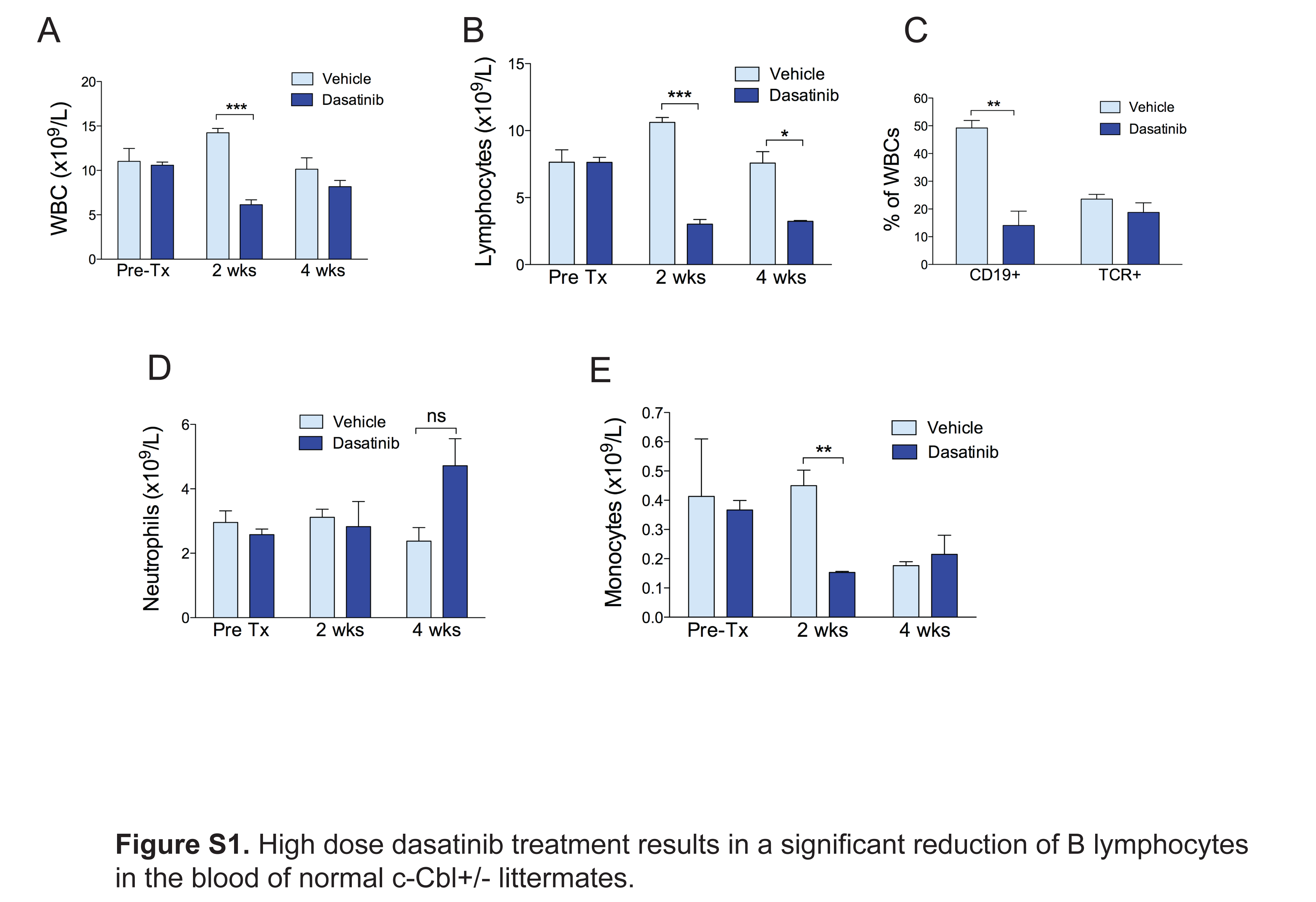

Supplement: Figure S1 — High dose dasatinib treatment results in a significant reduction of B lymphocytes in the blood of normal c-Cbl+/− littermates. c-Cbl+/− mice aged 8–9 months were dosed daily with 30 mg/kg (am) +50 mg/kg (pm) of dasatinib or vehicle, and bled before treatment (Pre-Tx), and after 2 and 4 weeks of treatment. Differential blood counts from 3 vehicle and 3 dasatinib treated mice were determined by Hemavet analysis. Shown are (A) total WBC numbers and (B) lymphocyte numbers. (C) WBCs were analyzed by flow cytometry to determine the percentage of B-lineage cells, by anti-CD19 staining, and the percentage of T cells, by anti-T cell receptor staining. (D) Numbers of neutrophils and (E) monocytes. By 4 weeks of dosing one of the dasatinib-treated mice had died, therefore only two dasatinib-treated mice were available for analysis at this time point. The results are expressed as means ± standard errors. *P<0.05, **P<0.01, ***P<0.001 using the unpaired Student’s t test. (TIF) [file pone.0094717.s001.tif]

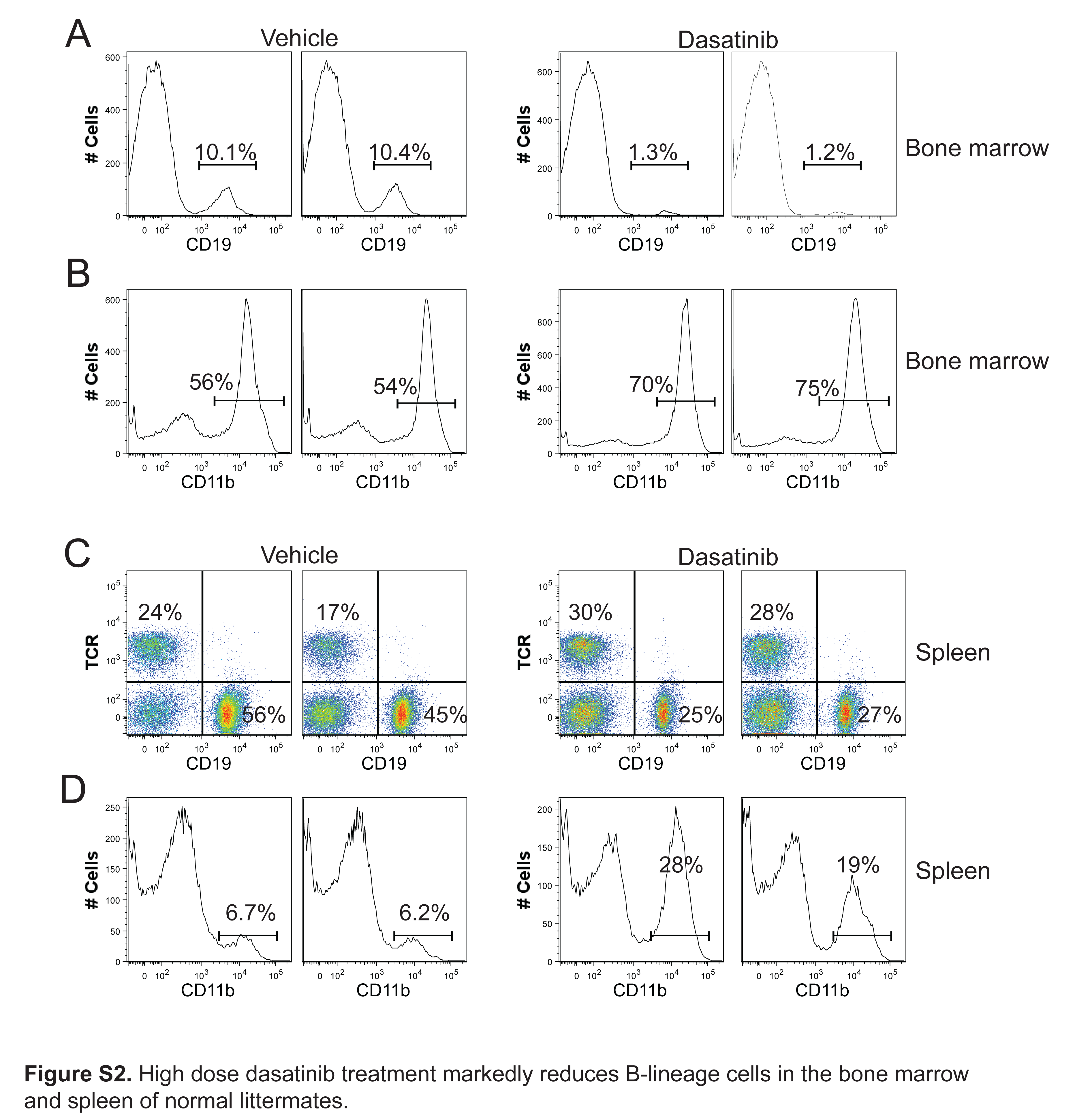

Supplement: Figure S2 — High dose dasatinib treatment markedly reduces B-lineage cells in the bone marrow and spleen of normal littermates. c-Cbl+/− mice aged 8–9 months were dosed daily with 30 mg/kg (am) +50 mg/kg (pm) of dasatinib or vehicle, and 2 mice from each group were analyzed after 4 weeks. Bone marrow cells were analyzed by flow cytometry to determine the percentage of (A) CD19+ B-lineage cells and (B) CD11b+ myeloid cells. The results showed that dasatinib caused a marked reduction in B-lineage cells and a corresponding increase in the proportion of myeloid cells. (C) Analysis of spleen cells from these mice showed that dasatinib caused a large reduction in CD19+ B cells, whereas T cells were not markedly affected. (D) CD11b analysis showed that dasatinib caused an increase in the proportion of myeloid cells in the spleen. (TIF) [file pone.0094717.s002.tif]
